# Correspondence article on the research protocol titled ‘ *Towards Health Equity and Transformative Action on tribal health (THETA) study to describe, explain and act on tribal health inequities in India: A health systems research study protocol*’ published in Wellcome Open Research in December 2019

**DOI:** 10.12688/wellcomeopenres.18425.1

**Published:** 2023-01-05

**Authors:** Rajnesh Kumar, Neha Dumka, Atul Kotwal

**Affiliations:** 1 Knowledge Management Division, National Health Systems Resource Centre, Delhi, Delhi, 110067, India; 2 Knoiwledge Management Division, National Health systems resource centre, Delhi, Delhi, 110067, India; 3 National Health Systems Resource centre, National Health Systems Resource centre, Delhi, Delhi, 110067, India

**Keywords:** Tribal health, Research ethics, Vulnerable population

## Abstract

Research on indigenous (Tribal) populations is a step towards understanding the various tribal health issues and challenges and paves the way for addressing these issues. However, such populations are categorised as vulnerable and marginalised according to National ethical guidelines by Indian council of medical research. Hence, adequate measures are needed to be ensured by researchers while undertaking any research involving tribal populations to safeguard the rights of research participants. The purpose of this correspondence is to initiate a discussion among the researchers to give due consideration to research ethics especially when the research is being conducted on vulnerable populations and take adequate safeguard measures as suggested by National ethical guidelines to protect the rights of study participants.

## Correspondence

We read with great interest the research protocol titled “ *Towards Health Equity and Transformative Action on tribal health (THETA) study to describe, explain and act on tribal health inequities in India: A health systems research study protocol*” published in Wellcome Open Research in December 2019 ^1^.

It is good to see that such a comprehensive research study is being conducted on tribal populations across India. The study is well designed with the potential to contribute to the body of literature on tribal health in India. Additionally, the proposed study intervention to enhance equity may provide insights on the design and pilot whether more such interventions are needed to address the health inequities among tribal communities in India. Methodology and study design though are scientifically sound however the comparison between tribal and non-tribal communities to understand health inequities and health-seeking behaviour may not give insights into the causal factors of inequities as they are more dependent on the socio-cultural contexts of communities rather than on sharing the same geographies ^2^. In this context researchers may also study the health differences within the sub-groups of indigenous community in the same geographies ^2^.

We would also like to highlight certain issues we observed in the study protocol. The title of the protocol specifies “Transformative action on tribal health inequities”, however, the collection of biological samples, genetic testing and saving samples in a biorepository seem to be inconsistent with the research title and their requirement to address the inequity issues is not understood, hence authors may revisit the title of the protocol.

This also raises serious ethical concerns related to informed consent and research on vulnerable participants as per the protocol published ^3^. During the entire research, investigators are obtaining recorded verbal consent from the participants after screening a video (replacing the Participant information sheet) to the participants, which raises the question as to how it will be ascertained that the participants have comprehended it. The low literacy rate among the tribal population has been cited as a reason for obtaining verbal consent which does not seem rational and is inconsistent with the Indian Council of Medical Research, (ICMR) National Ethical Guidelines for biomedical research on human participants in India ^3^. ICMR guidelines mandate that verbal consent can be obtained under exceptional circumstances with specific justifiable reasons and cannot be a routine practice in a research study ^3^. The protocol also does not provide any information on whether the participants are being informed that their genetic samples will be stored in a biorepository and also no specific purpose is mentioned as to why the genetic samples need to be stored in the biorepository and how it is related to a research study being conducted to describe, explain and act on health inequities among the population under study. ICMR guidelines on biological materials and biobanking mandate that written consent is necessary for collecting genetic samples for research purposes ^3^. Since the research involves a vulnerable population, there is no mention of additional safeguards and measures in place to protect the rights of research participants.

For a research project being carried out in India funded by a foreign agency the proposal must have been submitted to Health Ministry’s screening committee (HMSC) for approval, the protocol does not provide information on this aspect.

Research ethics are one of the major responsibilities of the researchers and they are expected to ensure that research is carried out in a responsible manner ensuring proper written consent ^4^. Whenever such issues are observed in the protocol, there are high chances of violating the rights of research participants during data collection. The investigators need to take the necessary steps and actions to safeguard participants’ rights given the above observations.

## Data Availability

No data are associated with this article.
