# Supplementary material for: Correspondence article on the research protocol titled ‘ Towards Health Equity and Transformative Action on tribal health (THETA) study to describe, explain and act on tribal health inequities in India: A health systems research study protocol’ published in Wellcome Open Research in December 2019
Source: Wellcome Open Res. 2023 Jan 5;8:3. [Version 1] doi: 10.12688/wellcomeopenres.18425.1 (PMC10568208; doi:10.12688/wellcomeopenres.18425.1)
Supplement: Peer review history

# Reviewer report: Reviewer response for version 1

**DOI:** 10.21956/wellcomeopenres.20432.r66656

**Published:** 2023-10-11

**Authors:** Malaisamy Muniyandi

**Affiliations:** 1 ICMR-National Institute for Research in Tuberculosis, Chennai, Tamil Nadu, India

The communication, a health systems research study protocol towards health equity and transformative action on tribal health (THETA) describes an important topic of health inequities among tribal communities in India and highlighting intervention to enhance equity. According to Census Report 2011, tribes constitute 8.6% of India’s population or about 104 million tribal individuals. Tribal population in India are geographically and culturally isolated and depict varied social as well as economic development. A number of commissions and committees have been appointed to identify the impediments to their development in the country and these committees have recommended a number of measures to eliminate the socio-economic imbalances existing in the tribal areas. Several programmes have been launched to remove these stumbling blocks to benefit the tribal population starting from the first Five Year Plan in 1951. In spite of these efforts, the tribes show a great variation in their economic, social, political, educational and health spheres. There are currently rare, systematically collected data on the availability of evidence for scale-up of newly recommended interventions for health improvements in tribal areas.

 In India, 705 ethnic groups are recognised as Scheduled Tribes. In central India, the Scheduled Tribes are usually referred to as *Adivasis*, which means Indigenous Peoples. Some tribal groups are still more vulnerable than others, Primitive Tribal Groups (PTGs) is a distinct category established by the Dhebar Commission in 1973. It was renamed as “Particularly Vulnerable Tribal Groups (PVTGs)” in 2006. In 2013, the Ministry of health and Family Welfare and the Ministry of Tribal Affairs, Government of India jointly constituted the expert committee on tribal health. This constitution of India recognises the special status of tribal people and provides safeguard to protect their rights and culture. However, it has been suspected that tribal people have poor health and unmet needs. Health and health care in tribal areas remained unsolved problems and there is a lack of data on tribal health. Universal Health Care and Universal Health Assurance are now accepted principles of India’s government policy. I wish to congratulate the Kumar and his team for bringing up these issues to limelight through their communication.

 I have also added some of the issues and actions taken by the Government of India that recognises the need for a roadmap for tribal health. The constitution led by the Ministry of Health and Family welfare and the Ministry of Tribal Affairs developed the national framework and roadmap to improve the access, content, quality and utilisation of health services among tribal population. It was also suggested to introduce new intervention strategies, formulate strategic guidelines and make recommendations on the requirement of additional resources. 

 With respect to ethics, provision of heath care to the tribal should be only on the principles of justices, rights and equity, not on the market principles. The health care delivery and content must be appropriate to the needs and culture of each tribe and its locality. Tribal people want to be autonomous so as to be able to preserve their identity, their way of life and culture. Capacity building efforts will be required to enable them to participate Gram Sabha, panchayat and districts. Health literacy among the tribe is also low, behaviour change communication, health education and use of information technology are important. Since the rural health statistics reveals huge gap in the health infrastructure and resources in tribal areas, tribal society have remained away from scientific knowledge about the causation of disease or the way to prevent or treat them. There is also need for a more holistic tribal health research plan that covers the gap in available and existing data on tribal health to generate knowledge and data.

Are arguments sufficiently supported by evidence from the published literature or by new data and results?

Partly

Is the conclusion balanced and justified on the basis of the presented arguments?

Partly

Is the rationale for commenting on the previous publication clearly described?

Yes

Are any opinions stated well-argued, clear and cogent?

Yes

Reviewer Expertise:

Health Economics, Health Technology Assessment, Infectious Disease, Tuberculosis, Tribal Health

I confirm that I have read this submission and believe that I have an appropriate level of expertise to confirm that it is of an acceptable scientific standard.

# Reviewer report: Reviewer response for version 1

**DOI:** 10.21956/wellcomeopenres.20432.r60496

**Published:** 2023-08-17

**Authors:** Prashant Kulkarni

**Affiliations:** 1 Department of Anthropology, Savitribai Phule Pune University, Pune, Maharashtra, India; 2 Centre for Infectious Diseases in India CIDI, Johns Hopkins Alliance for a Healthier World, Baltimore, Maryland, USA

**The correspondence highlights two important aspects of the research protocol - health inequities and the ethics of the research.**

 The tribal population represents 8.6% of the total population in India but there are huge variations among the tribal groups. So, before comparing the health inequities between tribal and non-tribal groups it is necessary to study or understand the health disparities across two tribal groups since these are influenced by socio-cultural and political factors.

 Health is a broader domain that may include disparities across social, economic, environmental, and structural disparities. If the protocol focuses on suggesting some intervention measures, then there should have been a clear mention of the reason for the collection of biological samples and genetic testing. The correspondence did not clearly specify if any details have been provided in the protocol regarding biological sample collection.

 In any research study, it is necessary to make it clear to the participants what procedure is going to happen and what is going to happen to them during the participation, and what is going to be the outcome. Though few of the participants may be illiterate the basic criteria for participation should be comprehension or understanding of the research subject. It’s always good to get consent in written (video-/audio recording) form to make it evident that the researchers have met the participants, however, the anonymity and confidentiality of the participants are maintained which may not be the case in video consenting. In case the participant is illiterate it is mandatory that the witness needs to be present who should not be related to the research team in any way and who the respondent can trust well. The witness attests that the consent information was accurately explained and that the participant apparently understood and informed consent was given freely. The witness should ensure that the participant has understood the topic well and the researcher should clarify the doubts if any. There also needs to be declared if there were no adverse events reported during biomedical sample collection.

Are arguments sufficiently supported by evidence from the published literature or by new data and results?

Partly

Is the conclusion balanced and justified on the basis of the presented arguments?

Partly

Is the rationale for commenting on the previous publication clearly described?

Yes

Are any opinions stated well-argued, clear and cogent?

Yes

Reviewer Expertise:

Public health, Health system research, Tribal health, communicable diseases

I confirm that I have read this submission and believe that I have an appropriate level of expertise to confirm that it is of an acceptable scientific standard.
